# Draft Genome Sequence of *Geobacillus kaustophilus* GBlys, a Lysogenic Strain with Bacteriophage ϕOH2

**DOI:** 10.1128/genomeA.00634-13

**Published:** 2013-08-15

**Authors:** Katsumi Doi, Kazuki Mori, Hindra Martono, Yuko Nagayoshi, Yasuhiro Fujino, Kosuke Tashiro, Satoru Kuhara, Toshihisa Ohshima

**Affiliations:** Microbial Genetic Division, Institute of Genetic Resources, Faculty of Agriculture, Kyushu University, Fukuoka, Japana; Department of Bioscience and Biotechnology, Faculty of Agriculture, Kyushu University, Fukuoka, Japanb; Division for Arts and Science, Faculty of Arts and Science, Kyushu University, Fukuoka, Japanc

## Abstract

*Geobacillus kaustophilus* strain GBlys was isolated along with the bacteriophage ϕOH2, which infects *G. kaustophilus* NBRC 102445^T^. Here we present a draft sequence of this strain’s genome, which consists of 216 contigs for a total of 3,541,481 bp, 3,679 predicted coding sequences, and a G+C content of 52.1%.

## GENOME ANNOUNCEMENT

The genus *Geobacillus* is comprised of moderately thermophilic, neutrophilic, aerobic or facultatively anaerobic, motile, spore-forming rods (1). *Geobacillus kaustophilus* was transferred to *Geobacillus* from the genus *Bacillus*, and its type strain, ATCC 8005 (NBRC 102445), was isolated from pasteurized milk (2). Among *G. kaustophilus* strains, the genome sequence has been reported only for strain HTA426, which was isolated from sea sediment from the Mariana Trench (3).

Recently, the features of bacteriophages that infect thermophilic bacteria have been attracting much attention. Among the *Geobacillus* phages reported so far (GVE1, GVE2, GBSV1, and D6E), only GVE2 is a lysogenic phage (4–7). The phage ϕOH2 (accession number AB823818), which infects *G. kaustophilus* NBRC 102445^T^, was isolated from the sediment of a hot spring. Its genome is integrated into the host genome, where it behaves as a prophage, producing the lysogenic strain *G. kaustophilus* GBlys. The genome sequence of strain GBlys may shed light on the mechanism by which ϕOH2 integrates into the host genome and may also be useful in comparative studies of the strains HTA426 and NBRC 102445^T^.

*G. kaustophilus* GBlys was collected from the center of a turbid plaque formed on a double-layer plate after infection of *G. kaustophilus* NBRC 102445^T^ with ϕOH2. Lysogenization of these cells was confirmed through induction experiments and PCR analysis. We prepared the GBlys sample for sequencing by growing the organism aerobically overnight at 55°C in growth medium 802 (Wako). Genomic DNA was then extracted and purified as described by Marmur (8), with some modification. Sequencing of the strain GBlys genome was accomplished using a combined approach with a 454 Genome Sequencer FLX system (Roche) and an Ion Torrent PGM sequencer (Life Technologies). The genomic DNA, which included a total of 3,541,481 bp, was sequenced using a whole-genome shotgun strategy that generated 827,782 reads and attained approximately 57-fold coverage. Assembly of all the reads using Newbler Assembler ver. 2.7 software resulted in 217 contigs with an *N*_50_ contig size of 61,981 bp. Annotation of the obtained scaffolds was performed using Glimmer 3.02 software and BLAST searches against a nonredundant protein sequence database (9).

The genome of strain GBlys has a G+C content of 52.1%. Annotation using the GTPS, RDP, and SILVA databases with tRNAscan-SE v.1.21, and with further manual inspection, revealed 2,154 predicted coding regions, 88 tRNA genes, and 10 rRNA genes. The ϕOH2 prophage genome is situated entirely astride contigs GBL0024 and GBL0110, and the host genes encoding GroES, GroEL, and transposase are located adjacent to the ϕOH2 prophage genome. A putative integrase gene is located at the terminal of the ϕOH2 prophage. There are no excisionase gene homologues, and attachment sites adjacent to the integrase gene are unlike those of phage λ (10). By comparing the genome sequences of *G. kaustophilus* strains GBlys and HTA426 using the BBH method with BLAST, we found that there are 2,943 orthologues with 98.52% identity.

### Nucleotide sequence accession numbers.

The *G. kaustophilus* GBlys genome sequence and annotation data have been deposited in the DDBJ/EMBL/GenBank under accession numbers BASG01000001 through BASG01000216.
